# Labdane-Type Diterpene and Two Flavones from *Salvia Sharifii* Rech. f. and Esfan. and their Biological Activities

**Published:** 2013

**Authors:** Mohammad Hossein Farjam, Abdolhossein Rustaiyan, Elham Ezzatzadeh, Amir Reza Jassbi

**Affiliations:** a *Department of Chemistry, Firoozabad Branch, Islamic Azad University, Firoozabad, Iran.*; b *Department of Chemistry, Science and Research Branch, Islamic Azad University, Tehran, Iran.*; c *Department of Chemistry, Ardabil Branch, Islamic Azad University, Ardabil, Iran.*; d *Medicinal and Natural Products Chemistry Research Canter, Shiraz University of Medicinal Sciences, Shiraz, Iran. *

**Keywords:** TLadanein, 6-hydroxy-5, 7, 4′-trimethoxyflavone, *Ent*-13-*epi*-manoyloxide, Antioxidant, Antimicrobial, Cytotoxic

## Abstract

Two flavones, ladanein and 6-hydroxy-5,7,4′-trimethoxyflavone and one labdane-type diterpene, *ent*-13-*epi*-manoyloxide, were isolated from an ethyl acetate-methanol extract of the aerial parts of *Salvia sharifii*. The compounds were purified using several chromatographic methods. Structural elucidation of the compounds was performed using their ^1^H and ^13^C-NMR data, EI mass and UV spectral data. The compounds have been subjected to antimicrobial, antioxidant and cytotoxic activity. The diterpene showed higher cytotoxic activity than the flavones while the later compounds were better antioxidants compared with the isolated diterpene.

## Introduction

*Salvia *(common name: sage), the largest genus of the Lamiaceae family, includes about 900 species. The word *Salvia *was derived from the Greek word ‘‘*Salvere*: healer, curative” and refers to the numerous medicinal applications of the plants of this family. These plants grow in the temperate and warmer zones of the world. Fifty-eight species are found in Iran, among which 17 are endemic (1). 

*Salvia sharifii *Rech. f. and esfan. is an endemic plant that grows wild in the south of Iran. Its Persian name is “Maryam-goli-e-jonoobi” and in the south of Iran, different preparations of this plant *e.g.*, decoctions, infusions and powders, are used as antiseptic, carminative, digestive and analgesic (2). To the best of our knowledge based on literature survey of different data banks, this is the first report on the chemical composition and biological activates on the aerial parts of this plant*. *The present research reports the chemical characterization of the structures of three compounds from the EtOAc/MeOH extract of the plant by ^1^H and ^13^C-NMR and Mass data. The antimicrobial effects of these compounds were evaluated against some pathogen strains of bacteria and fungi. The antioxidant activities of the compounds were also evaluated using 2, 2-diphenyl-1-picrylhydrazyl (DPPH) free radical scavenging assays. Finally, the cytotoxic activity of isolated compounds has been tested against two human cancer cell lines: colon carcinoma (TH-29) and breast ductal carcinoma (T47D).

## Experimental

*Plant material*

The aerial parts of the wild-growing *S. sharifii *were collected during the full flowering stage in May 2011 from the mountain areas of the Geno protected area in 30 Km west north of Bandar Abbas (Hormozgan Province, Iran) at an altitude of ca. 1800 m 27°29′ N and 55° 54′ E. It was identified by Dr. Mojtaba Asadollahi and a voucher specimen (NO. VC-19-8) was deposited at the Herbarium of Science and Research Branch, Islamic Azad University, Tehran, Iran. The aerial parts of the plant were air-dried at room temperature (25°C) in the shade for 5 days before the extraction.

*Extraction and isolation*

The separation process was carried out using several chromatographic methods (3). Ground aerial parts (500 g) were extracted with EtOAc : MeOH (1 : 1) (2 × 5 L) at room temperature for 3 days to give 45 g (8.3% yield) of the crude extract which was suspended in EtOH (300 mL) at 55ºC, diluted with H_2_O (259 mL) and extracted successively with *n*-hexane (3 × 650) and CHCl_3_ (3 × 450 mL). The CHCl_3_ extract on evaporation at reduced pressure furnished a residue (10 g) which was then subjected to column chromatography on silica gel (200 g) using *n*-hexane with increasing amounts of EtOAc (0-100%) up to EtOAc : MeOH (9 : 1). Fifty-one fractions were collected which were monitored by silica gel-TLC. Fractions 10-12 exhibited two spots on TLC were combined and after repeated CC-chromatography purification, yielded the *ent*-13-*epi*-manoyloxide (169 mg). After being monitored by TLC, fractions 33-35 were combined and recrystallized from hexane-EtOAc to remove the pigments impurities. Recrystallization from *n*-heptane : EtOAc (3 : 1) afforded 54 mg of pure 6-hydroxy-5,7,4’-methoxy-flavone. Fraction 36 was subjected to CC on silica gel (230-400 mesh) using hexane : EtOAc (7 : 1) to yield 70 mg of ladanein.

*Ent-13-epi-manoyloxide*

C_20_H_34_O, White powder, m.p. 70-72ºC, ^1^H-NMR (500 MHz, CDCl_3_): 0.79 (6H, br s, Me-18 and Me-19), 1.16 (3H, br s, Me-20), 1.29 (3H, s, Me-17), 1.44 (3H, s, Me-16) 5.05(1H, dd, *J *= 10.55, 1.30,H-15α), 5.23 (1H, dd, *J *= 17.55, 1.30, H-15β), 5.94 (1H, dd, J = 17.55, 10.95, H-14); ^13^C-NMR (125 MHz, CDCl_3_): C_14_ (145.8), C_15_ (111.2), C_8_ (76.7), C_13 _(77.0), C_8_ (76.7), C_5_ (61.5), C_9 _(56.0), C_1_ (44.95), C_3_ (44.4), C_7_ (41.9), C_12_ (39.6), C_4_ (33.3), C_10_(31.5), C_16_ (27.5), C_18_ (24.2), C_19_(22.6), C_17_ (21.4), C_20_ (20.5) C_2 _(18.4), C_11_ (18.3), C_6_ (15.3); MS (m/z) (rel.int.): 290 [M]^+^ (14), 275 [M-Me]^+^(7), 177 (88), 109 (68), 95 (84), 81 (80), 43 (100).

*6-hydroxy-6,7,4′-trimethoxyflavone*

C_18_H_16_O_6_, green crystals, m.p. 181-183ºC; 1H-NMR (500 MHz, CDCl_3_): 3.90 (3H, s, OMe-5), 3.93 (3H, S, OMe-7), 3.98 (3H, s, OMe-4′), 6.55 (1H, s, H-8), 6.59 (1H, s, H-3), 7.02 (2H, dd, *J *= 2.0, 9.0, H-3′ and H-5′), 7.85 (2H, dd, *J *= 1.9, 8.9, H-2′ and H-6′); ^13^C-NMR (125 MHz, CDCl_3_): OMe-5 (55.54), OMe-7 (56.30), OMe-4 (60.86), C_8_(90.55), C_3_ (104.12), C_10_ (106.12), C_5_′ and C_3_′ (114.51), C_1_′ (123.53), C_2_′ and C_6_′ (127.99),C_6_ (132.81), C_5_ (153.03), C_9_ (153.22), C_7_ (158.71), C_4_′ (162.6), C_2_ (164.00), C_4_ (182.65); MS (m/z) (rel.int.): 328 [M]^+^ (85), 313 [M-Me]^+^(85), 285 [M-Me-CO]^+^(37),), 167 (52), 149 (100), 69(80), 57 (60), 43 (44).

*Ladanein*

C_17_H_14_O_6_, White powder, m.p. 232-233ºC, 1H-NMR (500 MHz, CDCl_3_): 3.73 (3H, s, OMe-7), 3.92 (3H, s, OMe-4′), 6.86 (1H, s, H-8), 6.92 (1H, s, H-3), 6.93 (2H, d, *J *= 8.7, H-3′ and H-5′), 7.97 (2H, d, *J *= 1.9, 8.8, H-2′ ans H-6′), 12.93 (1H, s, OH); ^13^C-NMR (125 MHz, CDCl_3_): OMe-7(56.54), OMe-4 (60.04), C_8_(91.58), C_3_ (102.68), C_10_ (105.07), C_5_′ and C_3_′ (115.98), C_1_′ (121.01), C_2_′ and C_6_′ (128.58),C_6_ (131.85), C_9_ (152.09), C_5_ (152.63), C_7_ (158.62), C_4_′ (161.31), C_2_ (164.06), C_4_ (182.25); MS (m/z) (rel.int.): 313 [M]^+^ (29), 299 [M-Me]^+^(17), 279 (40), 214 (68), 167 (88), 149 (100), 57 (52), 43 (33).

*Antimicrobial activity*

Antimicrobial tests were carried out by the disc diffusion method (4) and MIC agar dilution assay (5). Isolated compounds were screened against six bacterial and three fungal strains. All microorganisms were obtained from the Persian type culture collection (PTCC), Tehran, Iran. *Antioxidant activity*

The antioxidant activates of the isolated compounds were evaluated using 2, 2 diphenyl-1-picrylhydrazyl (DPPH) free radical scavenging assays. This test was determined using a published DPPH radical scavenging activity assay method (6) with minor modifications.

*Cytotoxic activity*

The general method used for testing on antitumor properties of these compounds is the standard testing method that has been previously described in detail (7). Isolated compounds have been tested against two human cancer cell lines: colon carcinoma (TH-29) and breast ductal carcinoma (T47D).

## Results and Discussion

**Figure 1:**
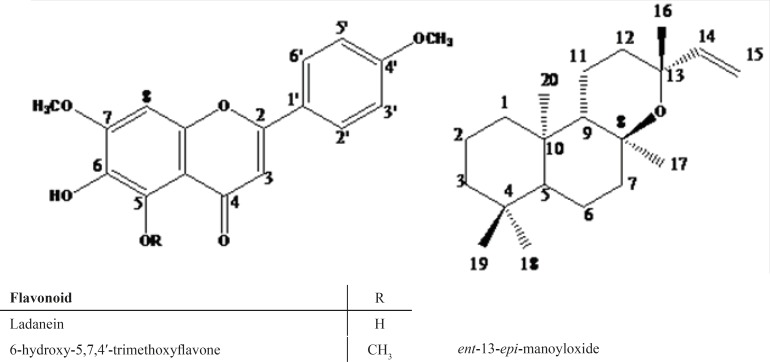
Isolated compounds from EtOAc/MeOH extract of *S.sharifii*

The isolated compounds were identified as ladanein, 6-hydroxy-5,7,4′ trimethoxyflavone, and ent-13-epi-manoyloxide (Figure 1). 

Structural elucidation was based on NMR and mass spectroscopic data, in comparison with those reported in the literature (8-10). The compound ladanein has the same oxidation pattern as that of 6-hydroxy-5,7,4′-trimethoxyflavone, except for the methoxy group in the fifth position. The ^1^H- and ^13^C-NMR for the isolated flavones and diterpene have been published previously. Ladanein is a known methoxylated flavone that found abundantly in *Salvia *species. It has been isolated from *S. hypoleuca*, *S. cyanescens*, *S. limbata *and *S. stenophylla *(11-14). 6-hydroxy-5,7,4′-trimethoxyflavone, containing three methoxyl at positions 5, 7 and 4′, is another methoxylated flavone but to the best of our knowledge, no similar finding in the *Salvia *genus has been reported by previous researchers. However, it has been previously isolated from *Orthosiphonstamineus *(15) and *Kaempferiaparviflora *(16). The third product isolated from the less polar fraction of *S. sharifii *extract was identified as ent-13-epi-manoyloxid, a labdane diterpene which seems to be a final metabolite of its biosynthetic branch. This compound has been isolated previously from *Guarea kunthiana *(17), *Chrysocephalum ambiguum *(18) and *Gibberella fujikuroi *(10) but, until now, not yet reported in the *Salvia *genus. In this study, we have reported for the first time that the aforementioned compounds have been extracted from *S. sharifii.*

We investigated the antimicrobial, antioxidant and cytotoxic activities of isolated compounds in laboratory standards. Results of antimicrobial tests showed that two isolated flavones remarkably inhibited the growth of all tested bacteria (especially against three Gram-negative bacteria) in terms of minimal inhibitory concentration (MIC) and zone of inhibition around the disc, while *ent*-13-*epi*-manoyloxide showed mildly activity against these microorganisms. Results of antimicrobial assessment were reported in Table 1.

**Table 1:** Antimicrobial activity of isolated compounds

**References** ^c^		***ent*** **-13-** ***epi*** **-manoyloxide**		**6-hydroxy-5,7,4′-trimethoxyflavone**		**Ladanein**		**Microorganism**
**DD**	**MIC**	**DD**	**MIC**	**DD**	**MIC**	**DD** ^b^	**MIC** ^a^	

0.1 ± 18.1	16	1.1 ± 6.1	256	0.82 19.0±	32	0.7 ± 18.1	32	*Escherichia coli *PTCC 1533
0.5 ± 15.3	8	0.5 ± 10.1	256	0.69 ± 14.5	128	0.5 ± 13.5	64	*Pseudomonas aeroginosa *PTCC1310
1.1 ± 18.6	32	1.5 ± 12.6	256	0.86 12.2±	64	0.9 ± 14	64	*Salmonella typhi *PTCC 1609
**Gram-positive bacteria**								
0.3 ± 16.1	64	0.5 ± 14.5	128	1.02 ± 8.3	128	0.5 ± 10.5	128	*Bacillus pumilus *PTCC 1319
0.5 ± 15.5	32	0.5 ± 10.6	256	0.3 ± 9.5	128	0.1 ± 11.7	128	*Kocuriavarians *PTCC 1484
0.1 ± 17.3	16	0.5 ± 8.9	128	2.37 ± 13.1	256	0.5 ± 12.6	128	*Listeria monocytogenes *PTCC1298
**Fungi**								
0.7 ± 25.5	64	1.1 ± 10.7	512	0.69 ± 6.4	512	0.3 ± 7.8	512	*Aspergillusflavus *PTCC 5006
0.9 ± 19.1	64	0.3 ± 13.1	256	1.8 6.7±	256	0.5 ± 7.5	512	*Candida glabrata *PTCC 5297
0.1 ± 27.0	32	1.0 11.0±	256	0.11 ± 6.4	256	0.2 ± 8.3	256	*Aspergillusniger *PTCC 5154

^a^ Minimum Inhibitory Concentration (μg/mL)

^b^Zone of inhibition (mm) in disc diffusion method , The values represent the mean of four experiments ± SD. All isolated compounds were tested at a concentration of 30 μL/disc.

^c^Ampicillin(5 μg/disc), gentamicin (10 μg/disc) and ketoconazole (100 IU) were used as references for Gram-positive, Gram-negative bacteria and fungus, respectively for disc diffusion method. In MIC assessment, Ampicillin, Tetracycline and Fluconazole were used as references for Gram-positive, Gram-negative bacteria and fungus, respectively.

 Both flavones were active against *Escherichia coli *with MIC of 32 μg/mL. This is particularly interesting from a medical point of view as this microbial agent is responsible for severe opportunistic infections. None of the isolated components showed significant activity against fungal microorganisms. We also screened the antioxidant activity of the isolated compounds from the aerial parts of *S. sharifii *by DPPH radical scavenging assay (Table 2).

**Table 2:** Antioxidant activity of isolated compounds by DPPH assay

**Compounds**	**Ladanein**	**6-hydroxy-5,7,4′-trimethoxyflavone**	***ent*** **-13-epi-manoyloxide**	**Quercetin**
DPPH IC_50 _(mg/mL)	5.3 ± 0.3	3.6 ± 0.1	16.2 ± 0.8	0.005 ± 0.001

Values represent the mean of three experiments ± SD. Quercetin was tested as a reference compound in the DPPH assay

 As expected for all phenolic compounds, ladanein and 6-hydroxy-5,7,4′-methoxy-flavone showed higher antioxidant activities (IC_50_ = 5.3 ± 0.3 and 3.6 ± 0.1 mg/mL respectively) in comparison with the non-phenolic diterpene, *ent*-13-*epi*-manoyloxide (IC_50_ = 16.2 ± 0.8 mg/mL). In cytotoxic activity, the effects of these compounds on the proliferative response of the HT-29 and T47D cell lines have been analyzed by treating the cells with different concentrations of the compounds in DMSO and decrease in cell lines proliferation were observed. *Ent*-13-*epi*-manoyloxide exhibit high cytotoxic activity on T47D (IC_50_ = 80.89 ± 4.05 μg/mL) and HT-29 (IC_50_ = 31.30 ± 1.32 μg/mL) while this property in isolated flavones is less (Table 3). 

**Table 3:** Cytotoxic activity of isolated compounds

**Compounds**	**IC**_50_ **(μg/mL) ** ^a^	
	**HT-29**	**T47D**
Ladanein	405.71 ± 13.26	573.32 ± 48.52
6-hydroxy-5,7,4′-trimethoxyflavone	461.63 ± 8.06	> 800
*ent*-13-*epi*-manoyloxide	31.30 ± 1.32	80.89 ± 4.05
Methotrexate	0.23 ± 0.2	0.16 ± 0.09

^a^Values represent the mean of 3-4 experiments ± SD. Methotrexate was tested as a positive control. Key to cell lines employed: colon carcinoma (HT-29) and breast ductal carcinoma (T47D

These results showed that this medicinal plant has the required potential to be used in aromatherapy and pharmacy.
